# Using the Preparation Phase of the Multiphase Optimization Strategy to Develop a Messaging Component for Weight Loss: Formative and Pilot Research

**DOI:** 10.2196/16297

**Published:** 2020-05-13

**Authors:** Angela Fidler Pfammatter, Sara Hoffman Marchese, Christine Pellegrini, Elyse Daly, Miriam Davidson, Bonnie Spring

**Affiliations:** 1 Department of Preventive Medicine Northwestern University Feinberg School of Medicine Chicago, IL United States; 2 Department of Exercise Science Arnold School of Public Health University of South Carolina Columbia, SC United States

**Keywords:** weight loss, body weight, text messaging, optimization, automation

## Abstract

**Background:**

Mobile messaging is often used in behavioral weight loss interventions, yet little is known as to the extent to which they contribute to weight loss when part of a multicomponent treatment package. The multiphase optimization strategy (MOST) is a framework that researchers can use to systematically investigate interventions that achieve desirable outcomes given specified constraints.

**Objective:**

This study describes the use of MOST to develop a messaging intervention as a component to test as part of a weight loss treatment package in a subsequent optimization trial.

**Methods:**

On the basis of our conceptual model, a text message intervention was created to support self-regulation of weight-related behaviors. We tested the messages in the ENLIGHTEN feasibility pilot study. Adults with overweight and obesity were recruited to participate in an 8-week weight loss program. Participants received a commercially available self-monitoring smartphone app, coaching calls, and text messages. The number and frequency of text messages sent were determined by individual preferences, and weight was assessed at 8 weeks.

**Results:**

Participants (n=9) in the feasibility pilot study lost 3.2% of their initial body weight over the 8-week intervention and preferred to receive 1.8 texts per day for 4.3 days per week. Researcher burden in manually sending messages was high, and the cost of receiving text messages was a concern. Therefore, a fully automated push notification system was developed to facilitate sending tailored daily messages to participants to support weight loss.

**Conclusions:**

Following the completion of specifying the conceptual model and the feasibility pilot study, the message intervention went through a final iteration. Theory and feasibility pilot study results during the preparation phase informed critical decisions about automation, frequency, triggers, and content before inclusion as a treatment component in a factorial optimization trial.

**Trial Registration:**

ClinicalTrials.gov NCT01814072; https://clinicaltrials.gov/ct2/show/NCT01814072

## Introduction

### Background

Mobile device use is ubiquitous in the United States: in 2018, 95% of adults owned a cell phone and 77% owned a smartphone [1,2]. Text messaging, or SMS, is a common feature of many users’ plans, with 81% of users indicating that they send or receive text messages as a part of regular phone use [3]. More than half of Americans look for health information on their mobile phone [4]. Thus, research on effectively using mobile systems to deliver health interventions has proliferated in recent years.

Messaging (also referred to as *texting*, *sending messages*, or *text messaging system*) affords many attractive features for mobile health interventions. Messaging systems can have broad reach using few resources compared with intervention components requiring consistent human labor or in-person delivery. Furthermore, message delivery can be systematically automated per predetermined decision rules, reducing the personnel costs associated with requiring an interventionist to monitor and trigger messages in real time. Text messages can be sent to the most basic of mobile phones at little cost, or the same content can be sent through a native smartphone app as a push notification at no cost to the recipient. Hence, mobile messaging can be implemented cost-efficiently on mobile devices in a variety of ways to fit the needs of the target population.

Although the first randomized controlled trial examining messaging as a health intervention was only conducted in 2005 [5,6], messaging has now been used in a variety of health interventions. Messages delivered by phones have shown positive effects on multiple health behaviors [6-9]. Yet, many questions about what content and at what frequency remain regarding the effective use of messaging to promote weight loss specifically [10]. These questions have become more compelling now that new technological innovations (such as real-time monitoring of continuous data from mobile sensors) make it possible to perform real-time data analytics as a basis for delivering just-in-time adaptive messaging interventions.

One common functionality of messaging is merely to deliver prompts or reminders about behaviors that need to be performed daily or multiple times per day. The complexity of reminders can vary from a once daily prompt (eg, to take medication [11]) to multiple daily prompts to take medications at particular times or to perform other behaviors (eg, physical activity [12]) multiple times daily. For more complex health targets such as weight loss that involve multiple behaviors that typically occur many times a day, thought needs to be invested in message timing and frequency [13]. If messages are not experienced as more helpful than burdensome, users may fail to attend to messages altogether. Customizing a messaging program to deliver messages flexibly, in a manner responsive to the needs of the user, could provide new opportunities to optimize positive effects of message interventions for more complex multiple behavioral demands.

### Message Tailoring

Personalized and tailored messages are established to be more effective than generic messages [6,8] in part because a message is perceived to be more relevant or actionable if tailored to be targeting the individual, some specific aspect of their behavior, or goal attainment. The question then becomes: What level of tailoring is necessary and on what variables should messages be tailored to garner a positive behavioral effect? Message tailoring for most behavioral interventions to date has been restricted to surface level features: for example, personalizing with name, gender, or another baseline variable that does not change over time [6,8,14]. For health behaviors that require recurrent messages over long periods of time, lightly tailored messages could become repetitive and ineffective. One strategy that is now possible involves tailoring messages in real time to respond to the user’s current state [15], thereby increasing the receptivity of the person to the message and its intended effect. Consistent with evaluations of other tailored interventions and learning theory, giving participants real-time feedback can improve engagement with the intervention, thereby producing improvement in desired health behaviors [16,17]. At this time, it is technically possible to create a messaging system for weight loss that could pull known data regarding self-monitoring consumption, physical activity, and weight to facilitate the participant’s performance of positive health behaviors over time. Therefore, we sought to leverage new technology capabilities of messaging to support weight loss as a potential component of a weight loss program, first by using the preparation phase of the multiphase optimization strategy (MOST).

### The Multiphase Optimization Strategy: Preparation Phase

**Figure 1:**
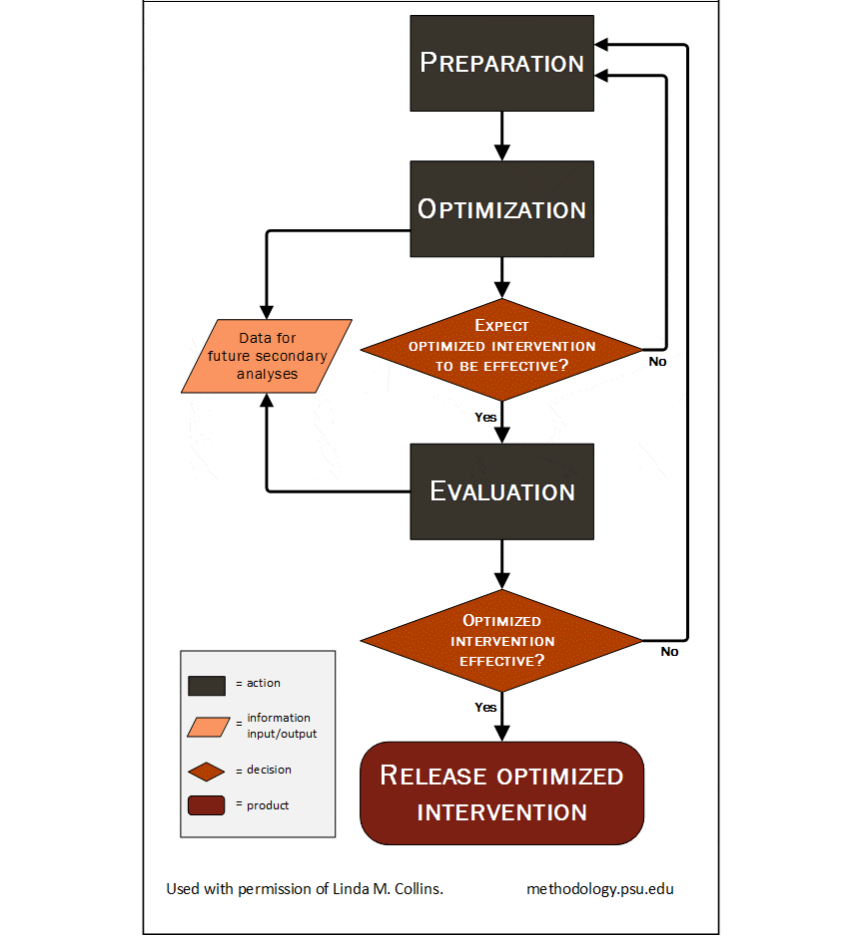
The multiphase optimization strategy (MOST).

MOST comprises several phases as depicted in Figure 1 [18] that support the design, assembly, and evaluation of a treatment package that meets optimization criteria, guided by some need as determined by an investigator. MOST provides a framework to systematically and efficiently improve interventions and thereby move intervention science forward. One use of the first phase, the preparation phase, of MOST is to engage in formative work and feasibility or pilot testing of treatment aspects, levels, or components before moving on to the optimization phase. The purpose of this paper is to describe the preparation phase of our project, when we engaged in a review of the literature and behavioral theory, conducted a pre-post feasibility pilot, and elicited user feedback and preferences to develop an automated, responsive, tailored messaging program to support weight loss as one component of a treatment package. By doing so, we ensure that the messaging component is based on sound behavioral theory, is feasible to deliver, and meets the needs of participants before implementing the component in a larger factorial trial (Optimization of Remotely Delivered INtensive Lifestyle Treatment for Obesity Study [Opt-IN]; ClinicalTrials.gov NCT01814072). Opt-IN, described in detail elsewhere [19,20], is a 6-month behavioral weight loss study comprising 32 experimental conditions.

## Methods

### Objectives

The first objective in the preparation phase of this work was to define a conceptual model to guide the message component development and testing. We did so in alignment with social cognitive theory. The second objective was to determine the feasibility of constructing and delivering messages to participants and the acceptability and preferences regarding the messages to inform implementation of the message component in the future optimization trial. Finally, we structured the logic and fully programmed the component to be tailored and automatically delivered with fidelity.

### Preparation Phase Part 1: Specifying a Conceptual Model

Social cognitive theory is one model for behavioral change that can be helpful in describing how a messaging intervention could contribute to weight loss [21]. Specifically, the provision of behavioral facilitation and supportive accountability via messaging could build self-efficacy, and the confidence about being able to perform a specific behavior despite the presence of challenges and barriers.

Behavioral facilitation, or the provision of instrumental and informational support, refers to supporting the individual with knowledge, skills, resources, or adjustments to the environment that can make unhealthy behaviors easier to change [22]. When an individual receives tips, directions, or aids in problem solving, they may perceive navigating difficult behavior changes as less overwhelming and vicariously learn how to perform self-regulatory behaviors [23]. Building the ability to do the behavior is an essential ingredient of self-efficacy.

*Supportive accountability* refers to a relationship of encouragement and accountability that is established between two individuals, such as participant and coach, and that has been demonstrated to enhance adherence to positive behavior change [24-26]. The supportive accountability model postulates that adherence is strengthened when participants and coaches maintain a therapeutic bond, wherein the expectations of being cared about and monitored throughout the intervention are clearly stated. The provision of verbal persuasion and performance feedback can enhance a sense of mastery and further build self-efficacy.

**Figure 2:**
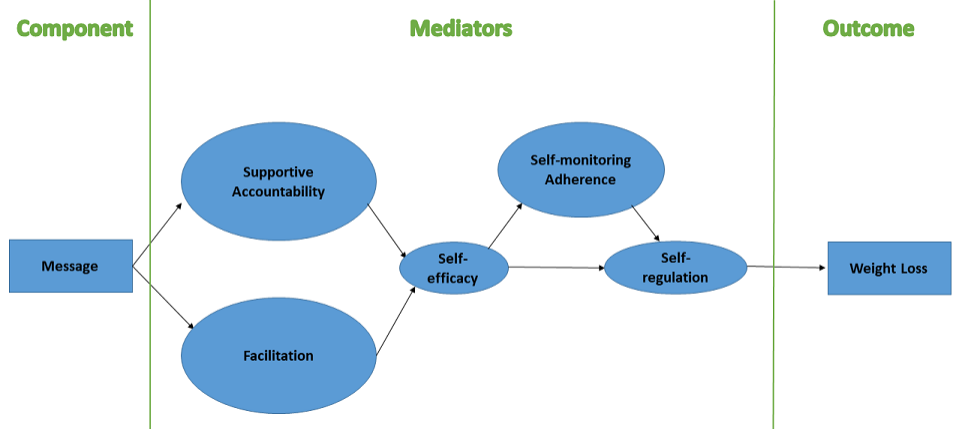
Optimization of Remotely Delivered INtensive Lifestyle Treatment for Obesity Study (Opt-IN) messaging conceptual model.

The proposed conceptual model (Figure 2) postulates that providing both behavioral facilitation and facilitation guide individuals toward experience with behavioral changes that increase self-efficacy. We hypothesize in our theoretical model that these two domains, facilitated by messages we send, will both produce increases in self-efficacy, thereby increasing self-regulation. Self-regulation, an internal process, involves the use of self-control, internal monitoring of behaviors in relation to a goal or value in the face of temptation, and is a well-established and important capacity that is strongly tied to success in weight loss [27]. When an individual gains self-efficacy for the health behaviors, the social cognitive model posits that self-regulation, or the internal capacity to independently regulate desires in favor of healthy behaviors, is strengthened.

### Preparation Phase Part 2: The ENLIGHTEN Feasibility Pilot Study

To test feasibility and acceptability of messaging and preliminary efficacy for producing weight loss, an 8-week weight loss pre-post feasibility pilot study was conducted using a commercially available smartphone app (ie, Lose It!), coaching calls, and text messaging. The purpose of this feasibility pilot study was to determine whether the messaging strategy was acceptable to participants and to determine the feasibility of messages. As such, the feasibility pilot study was not powered. The ENLIGHTEN study was approved by Northwestern University’s Institutional Review Board (STU00078810).

#### Participants

Participants were recruited from a large Chicago area employer. Enrollees had to be between 18 and 60 years of age, have a BMI between 25 and 40 kg/m^2^, weight <300 lb, be weight stable, and own an Android (Google Mobile Services) or iPhone (Apple) smartphone. Participants were excluded if they had unstable medical conditions, high risk for cardiovascular symptoms with physical activity, diabetes requiring insulin, Crohn’s disease, obstructive sleep apnea, active binge eating, substance abuse or dependence, or active suicidal ideation. Participants were also excluded if they took medication known to cause significant weight gain or loss, used an assistive device for mobility (eg, wheelchair, walker, cane), had been hospitalized for a psychiatric disorder within the past 5 years or could not read the study questionnaires. Female participants could not be pregnant, trying to get pregnant, or lactating. Each participant completed an informed consent process and selected the timing and frequency of text messages (up to 3× per day, including weekends) that were manually sent to them by a coach throughout the week. Text messages addressed the topics of diet, physical activity, and weight change. Each message either targeted the construct of supportive accountability (eg, “Way to get that exercise in this week!”) or facilitation (eg, “It’s a beautiful day, get out, and enjoy an after dinner walk!”).

## Results

### Results of Preparation Phase 2

The ENLIGHTEN feasibility pilot study enrolled 9 participants (6/9, 67% female; 5/9, 56% black; mean age 42.4 years, mean weight 197.2 lb, mean BMI 31.8 kg/m^2^). Participants who completed the intervention (n=8) lost an average of 3% of their initial body weight (range: +0.75 lb to −14.75 lb) and preferred to be sent an average of 1.8 texts per day, on 4.3 days of the week, with a range of 2 to 7 texts per week.

### Preparation Phase Part 3: Final Development of Message Treatment Component

The final stage of our preparation phase was to fully develop the message treatment component such that it could be implemented in the next phase, the optimization trial. To reduce the burden on staff and ensure fidelity of the treatment, we automated message sending by specifying programming logic for frequency and timing, content sent, and tailoring in response to user status.

### Automation of Messaging

One limitation of the feasibility trial to note was the significant staff burden required to manually send text messages to participants. As such, automation of sending messages was of paramount importance during further message program development. Automating, via a push notification protocol, not only reduces staff time but also enables the interventionist to maintain treatment fidelity due to the lack of human error that can produce untimely, missed, or poorly constructed text messages. Text messages are typically sent via SMS, which utilizes a 160-character restricted protocol. In contrast, we use Apple’s push notification service for iOS and Google Cloud Messaging service for Android. In style, length, and prominence, push notification messages are intended to be similar to SMS text messages. The advantages of the technology structure or architecture are 2-fold. First, neither participants nor study staff pays text message charges through their phone plan. Second, messages are integrated within the smartphone app to be used for self-monitoring in the optimization trial. Participants are alerted when a message arrives through content that pops up on either the iPhone’s lock screen or the Android’s notification bar. Clicking on the message opens the smartphone app and guides the participant to the message, providing an opportunity for the participant to continue engaging with the app after opening the message.

### Message Frequency and Triggers

Specific timing of the messages was structured to balance the message value with the burden that notifications place on the participant [28]. The feasibility pilot study revealed a frequency preference of 1 or 2 messages per day on average, but the number preferred varied widely. Due to the wide variability in preference, and given the Enlighten feasibility pilot study average of 1.8 texts per day on 4.3 days of the week (corresponding to approximately 7.7 texts per week), participants in the optimization trial chose what times were most convenient and least burdensome to receive a minimum *dose* of 7 text messages each week (Table 1). Timing choices included receiving a text at random times between 8 AM and 5 PM, only in the AM between 8 AM and noon, only in the PM between 12 PM and 5 PM, or a rotation where texts were alternately sent in the AM or PM. Participants could also choose whether to receive 2 bonus messages per week that were sent on their own varied schedule.

**Table 1:** Message preference schedules for participant selection.

Preference option^a^	Monday	Tuesday	Wednesday	Thursday	Friday	Saturday	Sunday
A	T1^b^	T2	T3	T4	T5	T6	T7
B	T1/T2	N/A^c^	T3/T4	N/A	T5/T6	N/A	T7
C	N/A	T1/T2	N/A	T3/T4	N/A	T5/T6	T7

^a^Schedule of text messages to be sent across a week.

^b^T: Text.

^c^N/A: not applicable.

**Figure 3:**
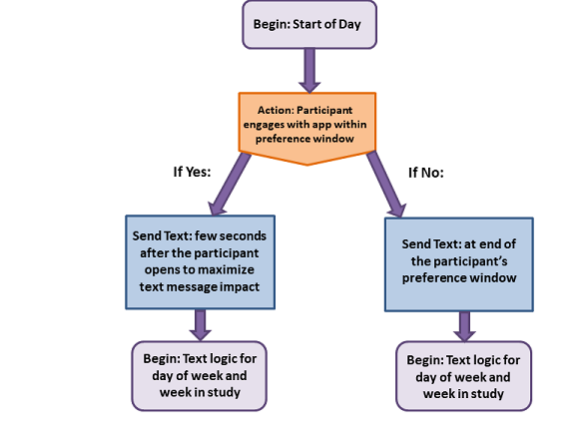
Logic structure for Optimization of Remotely Delivered INtensive Lifestyle Treatment for Obesity Study (Opt-IN) text message triggers.

The message trigger acted as the beginning of a logic structure that we hypothesize to increase likelihood of seeing the message (Figure 3), which supports fidelity of treatment receipt of our message component. If the participant opens the app during one of their preferred day and time windows, we infer that they are ready to receive a message, and thus a message will be sent (Figure 3). A slight delay is in place to avoid bombardment, giving the user time after they first open the app to complete a self-monitoring entry (eg, weight, food intake) before compiling and sending any pending messages. However, not all participants will interact with the app during their preference windows. To account for this, a message was automatically sent at the end of the preference period if no interaction with the smartphone app occurred. For example, if a participant’s preference is to receive one message every day in the morning (between 8 AM and 12 PM) and they have not opened the app at all during that window, a message is sent at midnight.

### Message Content

The content of all messages relates to behavioral facilitation or supportive accountability from the conceptual model in Figure 2. To make all messages relevant and engaging, a schedule of messages was created to target all aspects of weight loss and associated behaviors without becoming monotonous. Thus, participants received at least 7 messages per week, each with content from one of 7 behavioral categories: weight, physical activity, behavioral activation, adherence, physical activity goal attainment, calorie consumption goal attainment, or fat gram consumption goal attainment. The first message each week is from the weight category, which updates the participant on weight change over the previous week. The order of the remaining topics is different but predetermined each week for consistency across participants (Table 2). For example, a participant in week 2 of the study would receive a behavior activation message as their second text of the week, but someone in week 11 would receive a calorie consumption goal attainment text as their second text.

**Table 2:** Sample weekly text schedule with example content.

Weekly example	Monday	Tuesday	Wednesday	Thursday	Friday	Saturday	Sunday
Topic	Weight	MVPA^a^ goal attainment	Adherence	Behavioral activation	MVPA	Fat goal attainment	Calorie goal attainment
Message	You have lost X pounds in the past week! Way to go—remember to maintain these behavior changes as you continue with your weight loss!	Try to schedule in your activity! Make it a priority so you can meet those goals!	Focus on getting back on track with food entry on your phone in order to help you know your caloric and fat intake!	Adding in a small breakfast like a yogurt or an apple can make a big impact on your weight loss success!	Nice work exercising this week! Keep it up for the rest of the week.	If you're having trouble adding the right kinds of fat into your diet or recording your foods, don't be afraid to ask for suggestions on your next call.	It looks like you went over your calorie goal yesterday. Don't worry, slips are normal! Get back on track today.

^a^MVPA: moderate-vigorous physical activity.

### Tailoring in Response to User Status

**Figure 4:**
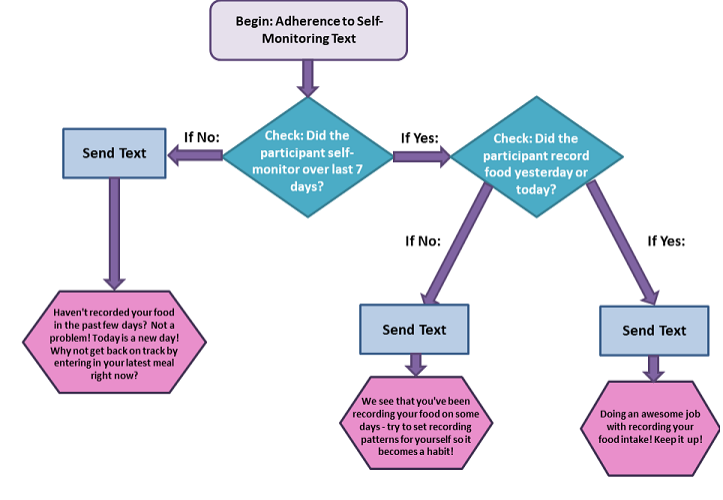
Optimization of Remotely Delivered INtensive Lifestyle Treatment for Obesity Study (Opt-IN) message tailoring: adherence to self-monitoring example.

To increase relevance and engagement, the text content is tailored to the individual’s personal progression toward study goal attainment (ie, weight loss, daily calorie intake, daily fat gram intake, and physical activity). As such, most messages delivered are based on what the participant has (or has not) self-monitored within the smartphone app. The texts are designed to encourage and reinforce positive participant behaviors, not to be negatively critical or discouraging. Hence, content was written to reinforce not only achieving goals, but also coming close to goal attainment, much like a human health coach might. For example, if a participant with a 1200 daily calorie goal enters 1220 calories, a calorie goal attainment text message would still reflect that they did well staying in the calorie range, rather than telling them they exceeded their daily calorie goal. Similarly, if a participant had not self-monitored food in the app on the day an adherence to self-monitoring text was to be sent, but had self-monitored food the prior day, the adherence to self-monitoring text would reinforce their recording on some days, while encouraging them to continue setting patterns (Figure 4).

Tailoring messages to make them feel both personalized and as if they are coming directly from the health coach was also considered an important factor in maintaining a strong therapeutic alliance between participant and their coach. Based on the assumption of this kind of social presence, we posit that the simulated human connection through messages created with this structure will be more efficacious than a *robot* or automated machine [6,24,29].

## Discussion

### Summary of Findings

As part of the MOST framework, critical preparation work was completed to develop a messaging protocol that was theoretically grounded, responsive to user feedback, easy to scale in a remotely delivered intervention, tailored to the individual’s current state, and feasible to deliver with fidelity in a factorial trial. Despite widespread use of messaging as a component of health behavior change interventions, information about the derivation of message content is often absent, leaving it unclear how and why the messages were developed and implemented in a particular way [6]. Furthermore, message interventions are often delivered as part of a treatment package and may not be able to be compartmentalized to test unique effects on outcomes due to overlapping function with other treatment components. Therefore, before embarking on an attempt to use a factorial design to optimize a treatment package for obesity, it was critical to spend time in the preparation phase to fully develop the messaging treatment component we wanted to test for effect on weight loss.

The development of our messaging component included designing messages based on theory, testing the feasibility and acceptability of the messages, and identifying participant preferences regarding message receipt. The resulting messages are personalized, timely, and relevant to the participant, which may increase the likelihood that the participant will respond in a desirable manner by maintaining or improving a targeted behavior. The preparation work also allowed us to create a component with potential for high treatment fidelity, a critical need in intervention trials and especially for factorial trials with a high number of randomized conditions. The resulting technology has the unique ability to deploy messages either in response to interaction with the smartphone app or based on the participant’s preferred schedule. This capitalizes on participant receptivity: when the individual is actively using the app, a time they are most likely thinking about their health behaviors. If an individual is not interacting with the study app, sending messages based on their preferred schedule may provide a real time-time helpful reminder to re-engage with target health behaviors to prevent a lapse.

### Artificial Intelligence

The features of our message component may very well be perceived as an artificially intelligent system. Messages are sent at times of high receptivity, with content that uses current participant status, and that responds in a way to support the self-efficacy of an individual much in the way a live health coach might. Human staff have availability constraints, cost a significant amount, and are prone to make errors in intervention delivery. Therefore, using human staff to coach individuals in a weight loss program has many barriers to scalability. The message program design described, if effective, may well be a first foray into artificial intelligent coaching that can provide just-in-time adaptive interventions. In sum, these essential preparatory activities supported the development of a theoretically sound, scalable, and low-cost treatment component that was feasible to deliver in our optimization trial design.

### Critiques and Strengths

One possible critique of our work is that it had a significant upfront cost to design and program sufficient to meet our requirements and confer a realistic and human feel. The overall cost might be worth time and effort downstream if it relieves staff time across enough participants over enough time. One benefit is that once programmed, as we have done, it can be scaled up and distributed widely with a relatively low maintenance cost, an important requirement set during our preparation phase. Comparatively, many intervention components are developed as part of multicomponent treatment packages, sometimes without critical preparatory work, and tested as such in a randomized controlled trial, the results of which are unable to reveal whether the component has any important or significant effect on the outcome. We believe the upfront cost of preparation in the context of MOST is warranted to develop rigorous, robust, and testable components.

### Future Work

Although weight loss programs that include a text messaging component have been effective [8,30-32], current evidence is not clear if it is an essential active component of a treatment package. In the next phase of this research, the optimization phase, the Opt-IN study will include messages as a factor that participants will be randomized to receive or not receive in a factorial research design. By using a factorial design, we will answer whether this type of messaging program provides a meaningful effect in a weight loss intervention package. It is critical to test the messaging component in this way because it provides significant advantages to investigators in that they are low-cost, easy-to-use, and can be delivered in real-time. This is an appealing alternative to costly human coaches who may not be able to deliver interventions at a time when the person needs it in everyday life [10,33-36].

Although our preparation phase work and the subsequent Opt-IN trial will make progress in optimizing a cost-contained treatment package for obesity, its limitation relevant to mobile messaging is that it will inform the utility of including messaging in a one-size fits all treatment package. One could also optimize weight loss by testing the use of message systems in stepped sequences as in a sequential multiple assignment randomized trial [37,38] or in specific just in time contexts as in a microrandomized trial [39]. The current lack of optimization is not limited to obesity treatment but has rather been unaddressed across various health interventions [7,15,40,41]. Thus, future research is needed to optimize all aspects of mobile messaging as a treatment component to fully realize its potential.

## Abbreviations

MOST: multiphase optimization strategy
NIDDK: National Institute of Diabetes and Digestive and Kidney Diseases
Opt-IN: Optimization of Remotely Delivered INtensive Lifestyle Treatment for Obesity Study
